# Effect of donor non-muscle myosin heavy chain (*MYH9*) gene polymorphisms on clinically relevant kidney allograft dysfunction

**DOI:** 10.1186/s12882-020-02039-6

**Published:** 2020-09-01

**Authors:** Joanna Pazik, Monika Oldak, Dominika Oziębło, Dominika Dęborska Materkowska, Anna Sadowska, Jacek Malejczyk, Magdalena Durlik

**Affiliations:** 1 Department of Transplantation Medicine, Nephrology and Internal Diseases, Medical University of Warsaw, 59 Nowogrodzka Street, 02-006 Warsaw, Poland; 2 Department of Histology and Embryology, Medical University of Warsaw, Warsaw, Poland; 3 Postgraduate School of Molecular Medicine, Medical University of Warsaw, Warsaw, Poland

**Keywords:** *MYH9*, Genetic biomarker, SNP, Kidney transplantation, Estimated glomerular filtration rate, Proteinuria

## Abstract

**Background:**

Despite its established association with chronic kidney disease (CKD) the role of myosin-9 (*MYH9*) gene variation on transplanted kidney function remains unknown. This study aimed at evaluating the effect of donor *MYH9* nephrogenic variants on renal allograft function within the first post transplantation year.

**Methods:**

In the longitudinal kidney transplant study 207 deceased donors were genotyped for previously known risk *MYH9* single nucleotide polymorphisms (SNPs). The predictor was *MYH9* high–risk variants status. The primary outcome was mean eGFR found in low vs. high risk *MYH9* genotypes between third and twelfth post-transplant month, the secondary outcome was the risk of proteinuria.

**Results:**

Distribution of genotypes remained in Hardy-Weinberg equilibrium. The T allele of rs3752462 (dominant model, TT or TC vs. CC) was associated with higher filtration rate (*P* = 0.05) in a multivariate analysis after adjusting for delayed graft function and donor sex. Two G alleles of rs136211 (recessive model, GG vs. GA or AA) resulted in doubling the risk of proteinuria (OR = 2.22; 95% CI = 1.18–4.37, *P* = 0.017) after adjusting for donor and recipient sex.

**Conclusion:**

Deceased donor kidneys of European descent harboring *MYH9* SNPs rs3752462 T allele show significantly superior estimated filtration rate while those of rs136211 GG genotype excessive risk of proteinuria. These findings, if replicated, may further inform and improve individualization of allocation and treatment policies.

## Background

Growing insight on how genetic variants of interest are linked to transplantation outcomes, give promise that screening genomes of organ recipients and donors would improve prediction of allograft longevity. In consequence novel targets for intervention, especially in organ allocation policies or immunosuppressive regimen adjustments would be provided [1, 2].

Nonmuscle myosin heavy chain II-A (NMMHC-IIA) is a universal contractile protein encoded by *MYH9* gene and expressed in muscle and non-muscle cells that engage in maintaining cell shape, adhesion, and division [3]. Despite growing evidence of the expression of NMMHC-IIA in the kidney tissue [4, 5], as well as its important function in podocytes cytoskeletal organization, cell adhesion, traction and motility [5–7], the role of *MYH9* variation in the pathogenesis of chronic kidney disease (CKD) remains unclear. Its functional mutations which cause the so-called *MYH9*-related disease, may affect the kidneys. This manifests clinically with persistent proteinuria and progressive loss of filtration leading to end-stage renal disease which histologically corresponds to segmental glomerular sclerosis [8, 9]. *MYH9* gene polymorphisms were associated with chronic kidney disease in genome wide association studies (GWAS) of Hispanic and European Americans. Studies conducted in the general Caucasian population have identified associations between intronic single nucleotide variants of *MYH9* and kidney function. O’Seaghdha et al. demonstrated an association of rs4821480 in the *MHY9/APOL1* region with the increased risk of early CKD in non-diabetic individuals of European ancestry [10] while Tavira et al. reported similar effect of rs3752462 in the adult Spanish population [11]. Pattaro found an association of SNPs within the *MYH9* gene and serum creatinine concentrations in three isolated European populations: rs2239784 and rs5756168 in MICROS cohort (The Genetic Study of three Population Microisolates in South Tyrol), rs136211 in VIS cohort (CROATIA-Vis study) and rs11089788 in the metaanalysis of three studied populations (MICROS, VIS and ERF cohort, Erasmus Rucphen Family study) [12].

In the peritransplant setting the effects of *MYH9* variants on renal allograft function might be augmented or mitigated by the exposure to inflammatory mediators, exo- or endotoxins, as well as immunosuppressive agents. At the same time ischemia was already identified as a second hit injury that reveals the effect of *MYH9* nephrogenic variants on GFR attrition, as in risk SNPs carriers with sickle cell anaemia, severe kidney ischemia triggered and enhanced secondary nephropathy [13, 14]. Moreover, in the mouse model of sickle cell anemia ischemic kidney injury modified *MYH9* gene and protein expression [15].

Our objective was to examine the association between selected SNPs and renal allograft function given as estimated glomerular filtration rate (the primary outcome) and risk of proteinuria (the secondary outcome). Our choice of the studied variants was based on literature data on *MYH9* SNPs known to correlate with CKD in Caucasians and we cite those in Table 1.

**Table 1:** Polymorphisms of the *MYH9* gene associated with CKD traits^†^. Data for variables with *P* < 0.05 are shown in bold

SNP	risk variant	European Americans non-diabetic [10]		European Americans [16]		Spanish Caucasians [11]		European isolated cohorts [5]							
								Micros^a^		Vis^b^		ERF^c^	Meta-analysis of Micros, VIS, ERF		
		eGFR< 60 ml/min		FSGS		eGFR< 60 ml/min		serum creatinine increment (mg/dl)							
		OR (95% CI)	p	OR (95% CI)	p	OR (95% CI)	p	b^#^ (SE)	p	b^#^ (SE)	p	b^#^ (SE)	p	b^#^ (SE)	p
rs3752462	T	–		**2.42 (1.17–5.04)**	**.01**	**1.99 (1.20–3.31)**	**.007**	–		–		–		–	
rs11089788	C	–		–		–		−0.0108 (0.0061)	.0782	−0.0204 (0.0143)	.1555	−0.0109 (0.0075)	.1465	**−0.0118 (0.0045)**	**.0089**
rs5756168	C	–		–		–		**0.0284 (0.0097)**	**.0034**	−0.0436 (0.0255)	.0875	0.0144 (0.0108)	.1839	0.0173 (0.0069)	.0127
rs2239784	T	–		–		–		**0.0309 (0.0142)**	**.0294**	−0.0069 (0.0269)	.7983	0.0158 (0.0172)	.3560	−0.0112 (0.0101)	.2674
rs136211	A	–		–		–		−0.0021 (0.0067)	.7502	**−0.0406 (0.0162)**	**.0123**	0.01240.0075)	.1009	0.0004 (0.0048)	.9414
rs4821480	G	**1.44 (1.15–1.80)**	**0.001**	**9.73 (1.07–463)**	**.02**	–		–		–		–		–	

^†^ table adapted from Liu L et at [16] supplemented with data from Pattaro et al. [12]

^#^linear regression coefficients (b) and SE, assuming an additive genetic model adjusted for sex and age

^a^MICROS cohort - The Genetic Study of three Population Microisolates in South Tyrol, Italy

^b^Vis cohort- CROATIA-Vis study

^c^ERF cohort, Erasmus Rucphen Family study, Netherlands

## Methods

### Study design and population

This was a longitudinal study in recipients of deceased donor kidney transplants who underwent transplantation at our institution; data was collected prospectively between January 2007 and December 2012. Genomic DNA samples used in this study were obtained form the KLINGEN kidney transplant cohort formed for the studies supported by grants of National Ministry of Science and Higher Education N N402 4266 33 and N N402 5668 40. These studies were approved by the local ethics committee and conducted following the Declaration of Helsinki.

The inclusion criteria were: 1) availability of a donor DNA specimen as well as donor and recipient clinical and immunological data and 2) transplantation procedure and post-transplant observation which were performed at our institution. The exclusion criteria were: 1) primary non-function of the graft, 2) donor’s age > 65 yrs., 3) recipient’s decision to continue post-transplant care at another center, 4) contralateral kidney graft from a donor already included in the study, 5) living donor transplantation, 6) non-Caucasian origin of the donor, and 7) retransplantation in a patient already included in the study.

Patients were accepted for the engraftment according to the uniformly applied national criteria for enlistment for kidney transplantation. If data was missing, it was assumed that the donor and recipient were of Caucasian origin.

### Study variables and outcome measures

The *MYH9* variants rs4821480, rs3752462, rs11089788, rs136211, rs5756168, and rs2239784 as well as clinical and peritransplant characteristics of implanted organs, donors, and recipients were considered as putative risk factors of transplanted kidney impaired filtration and proteinuria incidence.

Kidney allograft function, given as estimated glomerular filtration rate (eGFR) between third and twelfth post-implantation month was the primary outcome of the study that we assessed. Repeated estimations of GFR were performed with the Modification of Diet in Renal Disease (MDRD) 4-variable GFR equation based on serum creatinine concentrations at the 3rd, 6th, 9th, and 12th post-transplant month. Secondary outcome that we assessed was the incidence of proteinuria (given as dip-stic test) at the 3rd, 6th, 9th, and 12th post-transplant month.

Recognized clinical donor and recipient predictors of renal allograft function [17, 18] were included in the analyses: donor and recipient demographic data, donor cause of death, recipient type of primary kidney disease, recipient renal replacement treatment predating transplantation, HLA matching, Panel Reactive Antibodies (PRA), organ preservation technique (cold-storage vs pulsative perfusion), total ischemia time (TIT), delayed graft function (DGF) defined as haemodialysis requirement in the first post-transplant week, and acute rejection episodes within three post-implantation months. Donor HLA typing and cross-matching were performed at the central tissue typing laboratory while recipient HLA typing and PRA evaluations were performed locally.

### Post-transplant care and treatment

In the first months after transplantation, graft function was followed weekly, between the first and third month - monthly, and thereafter quarterly. In cases with delayed graft function, a surveillance biopsy was performed within the first two post-transplant weeks.

As a standard immunosuppressive regimen (in transplant recipients with low and intermediate immunological risk), combination of calcineurin inhibitors (CNI), steroids, and antiproliferative agents were used. High-risk kidney transplant recipients (retransplants or PRA > 20%) were additionally treated with either basiliximab, daclizumab, or rabbit antithymocyte globulin (rATG), those with PRA > 80% with rATG. The choice of CNI as well as antiproliferative agent was done at the discretion of the attending physician. Acute rejection episodes were diagnosed and graded according to the Banff ‘07 criteria [19] and treated as follows: borderline changes (3 × 250 mg methylprednisolone), Ia-IIa (3 × 500 mg methylprednisolone), and II-B or more advanced rejection (3–5 doses of 0.5 methylprednisolone, and rATG). Acute humoral rejection was diagnosed according to the Banff ‘07 standard criteria and treated with methylprednisolone, intravenous immunoglobulins (IVIG) and plasmapheresis.

### Statistical analysis

The genotype distributions were assessed for concordance with Hardy–Weinberg equilibrium using a χ^2^ goodness-of-fit test with significance level set to 5%. Fisher’s exact or Pearson’s X^2^ tests were used for the univariate analyses of the associations between categorical data. In order to compare mean values of quantitative variables between different groups, we used Mann-Whitney or Kruskal-Wallis tests.

The primary outcome was expressed as the estimated filtrations corresponding to *MYH9* variants in recessive, additive, and dominant models of inheritance. The secondary outcome was expressed as odds ratio of risk of proteinuria corresponding to *MYH9* variants in recessive, additive, and dominant models of inheritance. The quantitative measure of the effects sizes used in inference cause by factors were calculated using least square means (LSMEANS) or odds ratio (ODDS ratio) depending on the assumptions made within the linear mixed models.

For eGFR normal error distribution was used (MIXED Model), in the analysis of proteinuria binomial distribution was established (LOGISTIC Model).

Donor and recipient characteristics given in *Study variables* were considered as covariates.

All analyses were performed using SAS 9.4 (SAS, Cary, NC). We did not adjust the significance level of the *P* value for multiple hypothesis testing because we tested associations between eGFR and proteinuria on the basis of prior evidence linking studied SNPs with kidney disease (Table 1).

### Genotyping

Donor DNA was extracted from lymph node tissue samples with the standard phenol-chloroform extraction technique and was stored at − 20 °C. All tested SNPs were genotyped using custom TaqMan genotyping assays (Thermo Fisher Scientific, Waltham, MA, USA) according to the manufacturer’s instructions on the 7500 real-time PCR system and were analysed using 7500 system software (Applied Biosystems, Foster City, CA, USA). Each DNA sample was genotyped in two independent experiments, and positive and negative controls were included. Randomly selected samples representing different genotypes were sequenced using the Sanger method.

## Results

**Fig. 1:**
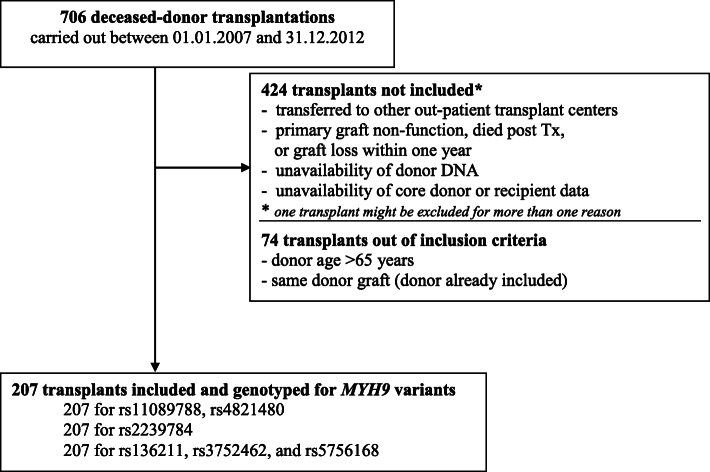
Study framework and patient recruitment

During the study period 359 deceased donors were identified. Out of consecutive 706 kidney-only transplantations, 207 organs were finally included in the analyses. Figure 1 shows the flowchart of the study sample selection.

Baseline characteristics of study grafts, donors, and recipients are shown in Table 2A. The genotyping success rate was 100% (207/207) for rs3752462, rs136211, rs5756168, rs11089788, rs2239784, and rs4821480. The genotypes distributions remained in concordance with Hardy–Weinberg equilibrium (as shown in Table 2B).

**Table 2:** Baseline characteristics of the 207 kidney transplants included in the study ^a^

**A. Recipients**				
Women, n (%)	83 (40.1)			
Age at transplantation (years)	48.1 ± 13.8 (19.0–77.0)			
Last PRA > 20%, n (%)	19 (8.3)			
Retransplants, n (%)	41 (19.8)			
HLA mismatch, n (%)				
0–2	65 (31.7)			
3–4	115 (56.1)			
5–6	25 (12.2)			
Delayed Graft Function^bc^ (DGF), n (%)	58 (34.0)			
Primary kidney disease, n (%)				
diabetes	21 (10.1)			
ADPKD	34 (16.4)			
GN	83 (40.1)			
hypertensive nephropathy	12 (5.8)			
other or unknown	57 (27.5)			
Dialysis before Tx (years)	4.6 ± 5.1 (0–31.3)			
Preemptive Tx, n (%)	3 (1.4)			
Induction with ATG or aIL2, n (%)	64 (31.5)			
Cyclosporine A, n (%)	53 (25.6)			
Tacrolimus, n (%)	154 (74.4)			
Mycophenolic acid preparation, n (%)	205 (99.0)			
Acute rejection within 1 yr, n (%)	35 (16.9)			
**B. Transplanted kidneys**				
Donor sex (woman), n (%)	83 (40.3)			
Donor age (years)	44.3 ± 13.9 (13.0–65.0)			
Donor cause of death^c^, n (%)				
stroke	110 (60.8)			
trauma	63 (34.8)			
other	8 (4.4)			
Preservation with pulsative perfusion, n(%)	126 (61.2)			
TIT^d^ (hours)	26.8 ± 8.1 (5.8–50.5)			
Donor *MYH9* SNP	n (%)	n (%)	n (%)	HWE p
rs11089788	AA: 55 (26.6%)	AC:105(50.7%)	CC: 47(22.7%)	0.707
rs136211	AA: 21 (10.1%)	AG: 91 (44%)	GG: 95(45.9%)	0.908
rs2239784	CC: 187 (90.3%)	CT: 20 (9.7%)	TT: 0 (0%)	0.442
rs3752462	CC: 112 (54.1%)	CT: 79(38.2%)	TT: 16 (7.7%)	0.580
rs5756168	CC: 3 (1.4%)	CT: 39(18.8%)	TT: 165(79.7%)	0.691
rs4821480	GG: 1(< 1%)	GT: 27(13%)	TT: 179(86.5%)	0.987

^a^ quantitative variables are expressed as mean ± SD (range);

^b^Due to missing data, the number of cases does not always add up to 207

^c^Missing data for 37 transplants

^d^ Missing data for 40 transplants

### Outcomes

The overall baseline eGFR (at the 3rd post-transplant month) was 46.91 ± 16.33 and increased to 49.75 ± 17.59, 50.25 ± 16.84 and 50.58 ± 17.79 ml/min/1.73 m^2^ at the 6th, 9th, and 12th post-transplant month, respectively (supplementary Figure 1). Univariate analyses performed for the three inheritance models revealed no associations between the genetic variants and eGFRs across the first post-transplant year (Table 3).

**Table 3:** Transplanted kidney eGFR at third and twelve post-transplant months per *MYH9* risk variant in additive, recessive and dominant models

			eGFR month 3				eGFR month 12			
SNP	genotype	n	Mean ± SD	Median	Range	p	Mean ± SD	Median	Range	p
rs11089788	AA	55	46.6 ± 18.6	42.2	15.5–118		49.0 ± 16.9	44.8	19.1–98.6	
	AC	105	47.8 ± 16.6	45.2	19.1–98.6	0.535^AAvsAC^	52.3 ± 19.7	51.6	19.5–162.9	0.224 ^AAvsAC^
	CC	47	45.4 ± 12.6	44.1	22.2–78.1	0.627 ^ACvsCC^	48.7 ± 13.8	48.4	12.2–84.2	0.351 ^ACvsCC^
rs11089788	AA	55	46.6 ± 18.6	42.2	15.5–118		49.0 ± 16.9	44.6	19.1–96.8	
	AC + CC	152	47.0 ± 15.5	44.6	19.1–96.8	0.574 ^AAvsC^	51.1 ± 18.1	50.8	12.2–162.9	0.273 ^AAvsC^
rs11089788	CC	47	45.5 ± 12.6	44.1	22.2–78.1		48.7 ± 13.8	48.4	12.2–84.2	
	AA+AC	160	47.4 ± 17.3	44.4	15.5–118	0.820 ^CCvsA^	51.1 ± 18.8	48.7	19.1–162.9	0.692 ^CCvsA^
rs136211	AA	21	49.4 ± 12.9	46.8	29.0–74.9		52.4 ± 14.4	51.7	22.4–84.2	
	AG	91	47.7 ± 14.8	46.3	21.3–91.3	0.467 ^AAvsAG^	50.7 ± 14.8	51.7	12.2–86.2	0.806 ^AAvsAG^
	GG	95	45.6 ± 18.3	41.2	15.5–118	0.123 ^AGvsGG^	50.0 ± 21.0	44.8	20.6–162.9	0.139 ^AGvsGG^
rs136211	AA	21	49.4 ± 12.9	46.8	29–74.9		52.4 ± 14.4	51.7	22.4–84.2	
	AG + GG	186	46.6 ± 16.7	43.6	15.5–118	0.208 ^AAvsG^	50.4 ± 18.2	48.4	12.2–86.2	0.377 ^AAvsG^
rs136211	GG	95	45.6 ± 18.3	41.2	15.5–118		50.0 ± 21.0	44.8	20.6–162.9	
	AG + AA	112	48.0 ± 14.4	46.6	21.3–91.3	0.064 ^GGvsA^	51.0 ± 14.7	51.7	12.2–86.2	0.085 ^GGvsA^
rs3752462	CC	112	45.8 ± 16.6	41.3	15.5–118		49.1 ± 15.9	47.3	19.1–91.3	
	CT	79	49.1 ± 16.1	47.3	19.1–91.3	0.077 ^CCvsCT^	52.4 ± 19.8	51.3	19.1–162.9	0.260 ^CCvsCT^
	TT	16	44.2 ± 15.1	42.1	27.7–83.7	0.208 ^CTvsTT^	52.0 ± 20.2	50.0	12.2–99.2	0.992 ^CTvsTT^
rs3752462	CC	112	45.8 ± 16.6	41.3	15.5–118		49.1 ± 15.9	47.3	19.1–91.3	
	CT + TT	95	48.3 ± 16.0	46.3	19.1–91.3	0.145 ^CCvsT^	52.3 ± 19.8	51.2	12.2–162.9	0.236 ^CCvsT^
rs3752462	TT	16	44.2 ± 15.1	42.1	27.7–83.7		52.0 ± 20.2	50.0	12.2–99.2	
	CC + CT	191	47.1 ± 16.4	44.6	15.5–118	0.461 ^TTvsC^	50.5 ± 17.6	48.6	19.1–162.9	0.709 ^TTvsC^
rs5756168	CC	3	53.2 ± 6.0	55.8	46.3–57.3		58.5 ± 22.5	49.2	42.2–84.2	
	CT	39	48.3 ± 13.8	47.8	22.2–86.7	0.366 ^CCvsCT^	51.0 ± 12.9	53.0	23.9–77.9	0.771 ^CCvsCT^
	TT	165	46.5 ± 17.0	43.5	15.5–118	0.254 ^CTvsTT^	50.3 ± 18.7	47.9	12.2–162.9	0.357 ^CTvsTT^
rs5756168	CC	3	53.2 ± 6.0	55.8	46.3–57.3		58.5 ± 22.5	49.2	42.2–84.2	
	CT + TT	204	46.8 ± 16.4	44.2	15.5–118	0.257 ^CCvsT^	50.5 ± 17.8	48.6	12.2–162.9	0.560 ^CCvsT^
rs5756168	TT	165	46.5 ± 17.0	43.5	15.5–118		50.3 ± 18.7	47.9	12.2–162.9	
	CC + CT	42	48.7 ± 13.4	48.4	22.2–86.7	0.169 ^TTvsC^	51.5 ± 13.6	52.7	23.9–84.2	0.298 ^TTvsC^
rs2239784	CC	187	46.5 ± 15.6	44.3	15.5–96.8		50.4 ± 18.0	48.8	12.2–182.9	
	CT	20	50.9 ± 21.9	46.8	27.7–118	0.569 ^CCvsCT^	52.6 ± 15.6	47.1	32.3–99.2	0.614 ^CCvsCT^
	TT	0								
rs2239784	CC	187	46.5 ± 15.6	44.3	15.5–96.8		50.4 ± 18.0	48.8	12.2–182.9	
	CT + TT	20	50.9 ± 21.9	46.8	27.7–118	0.569 ^CCvsT^	52.6 ± 15.6	47.1	32.3–99.2	0.614 ^CCvsT^
rs2239784	TT	0								
	CC + CT	207	46.9 ± 16.3	44.3	15.5118		50.6 ± 17.8	48.6	12.1162.8	
rs4821480	GG	1	51.6		–		55.8±			
	GT	27	51.8 ± 17.7	49.5	27.5–91.3		54.5 ± 18.3	51.4	30.1–99.2	
	TT	179	46.1 ± 16.1	44.1	15.5–118	0.126 ^GTvsTT^	50.0 ± 17.7	48.4	12.2–162.9	0.337 ^GTvsTT^
rs4821480	GG	1	51.6				55.8			
	GT + TT	206	46.9 ± 16.4	44.3	15.5118		50.6 ± 17.8	48.6	12.2–162.9	
rs4821480	TT	179	46.1 ± 16.1	44.1	15.5–118		50.0 ± 17.7	48.4	15.5–118	
	GG + GT	28	51.8 ± 17.4	49.8	27.5–91.3	0.108 ^TTvsG^	54.6 ± 18.0	51.9	30.1–99.2	0.295 ^TTvsG^

Proteinuria occurred in 14.7% at 3rd, 11.8% at 6th, 13.9% at 9th, and in 14.1% at 12th post-transplant months Univariate analysis identified a trend for excessive risk if proteiunuria in rs136211 GG kidneys: this genotype nearly doubled incidence of positive protein dip-stick test within first post-transplant year in opposition to A allele (OR = 1.96; 95% CI = 1.00–3.83, *P* = 0.056, Table 4, supplementary Figure 2).

**Table 4:** Proteinuria incidence between third and twelve post-transplant months per *MYH9* risk variant in additive, recessive and dominant models: Data for variables with P < 0.05 are shown in bold

			proteinuria month 3				proteinuria month 12				proteinuria between 3rd to 12th months			
SNP	genotype	n	Incidence (%)	OR	95% CI	P	Incidence (%)	OR	95% CI	P	Incidence (%)	OR	95% CI	P
rs11089788	AA	55	11.1				13.0				27.8		–	
	AC	105	15.5	1.47	0.54–4.01	0.629^ACvAA^	13.5	1.04	0.39–2.76	1.000 ^ACvAA^	26.0	0.91	0.43–1.92	0.850 ^ACvAA^
	CC	47	17.0	1.11	0.44–2.82	0.814 ^CCvAC^	17.0	1.32	0.51–3.40	0.621 ^CCvAC^	30.4	1.24	0.58–2.69	0.690 ^CCvAC^
rs11089788	AA	55	11.1		–		13.0		–		27.8		–	
	AC + CC	152	16.0	1.52	0.59–3.96	0.503 ^CvAA^	14.6	1.14	0.46–2.85	1.000 ^CvAA^	27.4	0.98	0.49–1.97	1.000 ^CvAA^
rs11089788	CC	47	17.0		–		17.0		–		30.4		–	
	AA+AC	160	14.0	1.26	0.52–3.05	0.609 ^CCvA^	13.3	1.34	0.55–3.25	0.485 ^CCvA^	26.6	1.21	0.58–2.48	0.707 ^CCvA^
rs136211	AA	21	9.5		–		9.5		–		25.0		–	
	AG	91	8.8	0.92	0.18–4.66	1.000 ^AGvAA^	12.1	1.31	0.27–6.39	1.000 ^AGvAA^	21.1	0.80	0.26–2.49	0.766 ^AGvAA^
	GG	95	**21.7**	**2.88**	**1.20**–**6.94**	**0.023** ^**GGvAG**^	17.2	1.51	0.66–3.46	0.406 ^GGvAG^	34.4	1.96	1.01–3.83	0.067 ^**GGvAG**^
rs136211	AA	21	9.5		–		9.5		–		25.0		–	
	AG + GG	186	15.3	1.72	0.38–7.78	0.745 ^GvAA^	14.7	1.63	0.36–7.42	0.745 ^GvAA^	27.8	1.15	0.40–3.34	1.000 ^GvAA^
rs136211	GG	95	**21.7**		–		17.2		–		21.8		–	
	AG + AA	112	**8.9**	**2.83**	**1.25**–**6.41**	**0.016** ^**GGvA**^	11.6	1.58	0.72–3.49	0.315 ^**GGvA**^	34.4	1.88	1.00–3.56	0.056 ^**GGvA**^
rs3752462	CC	112	15.4		–		15.3		–		29.0		–	
	CT	79	14.1	0.90	0.39–2.04	0.838 ^CTvCC^	12.8	0.81	0.35–1.88	0.678 ^CTvCC^	26.9	0.90	0.47–1.73	0.869 ^CTvCC^
	TT	16	12.5	0.87	0.17–4.37	1.000 ^TTvCT^	12.5	0.97	0.19–4.92	1.000 ^TTvCT^	20.0	0.68	0.17–2.64	0.752 ^TTvCT^
rs3752462	CC	112	15.4		–		15.3		–		29.0		–	
	CT + TT	95	13.8	0.88	0.40–1.92	0.844 ^TvCC^	12.8	0.81	0.36–1.79	0.689 ^TvCC^	25.8	0.85	0.46–1.59	0.637 ^TvCC^
rs3752462	TT	16	12.5		–		12.5		–		20.0		–	
	CC + CT	191	14.9	0.82	0.18–3.79	1.000^TTvC^	14.3	0.86	0.18–3.98	1.000 ^TTvC^	28.1	0.64	0.17–2.36	0.764 ^TTvC^
rs5756168	CC	3	33.3		–		33.3		–		33.3		–	
	CT	39	5.1	0.11	0.01–1.76	0.204 ^CTvCC^	7.7	0.17	0.01–2.42	0.265 ^CTvCC^	18.4	0.45	0.04–5.71	0.488 ^CTvCC^
	TT	165	16.7	3.70	0.84–16.28	0.077 ^TTvCT^	15.3	2.17	0.62–7.61	0.304 ^TTvCT^	29.6	1.86	0.76–4.52	0.224 ^TTvCT^
rs5756168	CC	3	33.3		–		33.3		–		33.3		–	
	CT + TT	204	14.4	0.34	0.03–3.84	0.359 ^TvCC^	13.9	0.32	0.03–3.67	0.369 ^TvCC^	27.4	0.75	0.07–8.50	1.000 ^TvCC^
rs5756168	TT	165	16.7		–		15.3		–		29.6		–	
	CC + CT	42	7.1	2.60	0.75–9.03	0.147 ^TTvC^	9.5	1.72	0.56–5.25	0.458 ^TTvC^	19.5	1.73	0.74–4.03	0.241 ^TTvC^
rs2239784	CC	187	15.2		–		15.1		–		28.7		–	
	CT	20	10.0	0.62	0.14–2.82	0.744 ^CTvCC^	5.0	0.29	0.04–2.29	0.320 ^CTvCC^	15.8	0.46	0.13–1.66	0.289 ^CTvCC^
	TT	0			–				–				–	
rs2239784	CC	187	15.2		–				–		28.7		–	
	CT + TT	20	10	0.62	0.14–2.82	0.744 ^TvCC^			–	TvCC	15.8	0.46	0.13–1.66	0.289 ^TvCC^
rs2239784	TT	0			–				–				–	
	CC + CT	207	14.7		–				–				–	
rs4821480	GG	1	0		–		0		–		0		–	
	GT	27	11.1		–	1.000 ^GTvGG^	18.5		–	1.000 ^GTvGG^	33.3		–	
	TT	179	15.4	1.50	0.41–5.15	0.773 ^TTvGT^	13.6	0.69	0.24–2.00	0.553 ^TTvGT^	26.6	0.72	0.30–1.73	0.490 ^TTvGT^
rs4821480	GG	1	0		–		0		–		0		–	
	GT + TT	206	14.8		–		14.2		–		27.5		–	
rs4821480	TT	179	15.3		–		13.6		–		26.6		–	
	GG + GT	28	10.7	1.51	0.43–5.35	0.774^TTvG^	17.8	0.72	0.25–2.08	0.561 ^TTvG^	33.0	0.72	0.30–1.73	0.490 ^TTvG^

At third post-transplant month it occurred in 8.8% vs 21.7%, in sixth in 8% vs 16.3%, in ninth 10.9% vs 17.6% and at 1 year in 11.6% vs 17.2% in allele A vs GG kidneys, respectively. Overall at least one episode of proteinuria occurred in 21.8% of patients engrafted with organs carrying A allele while in 34.4% carrying GG genotype.

### Multivariate models

**Fig. 2:**
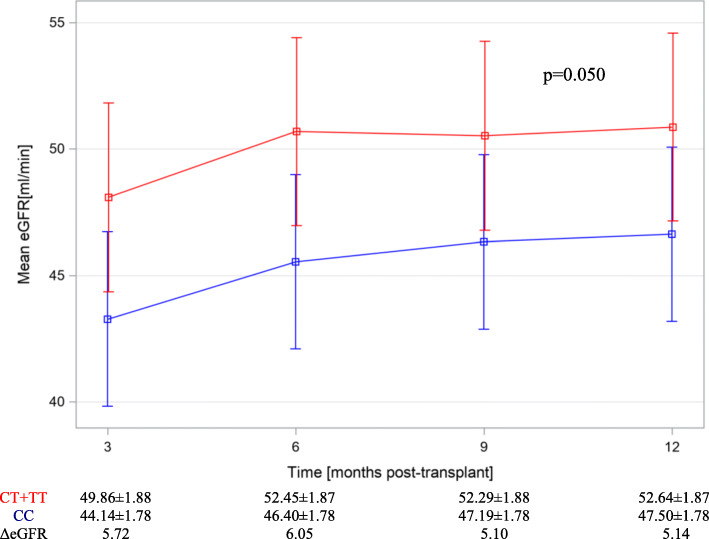
Mean estimated filtration rate (eGFR MDRD) within first post implantation year depending on rs3752462 genotype (CT or TT vs CC, four time-points model) after adjustment for DGF and donor gender

In the multivariate prediction model of eGFRs throughout first posttransplant year, a significantly higher filtrations were found for allele T of rs3752462 (dominant model) after adjusting for delayed graft function and donor sex, no allele T-by-time interaction effect was found (Table 5A). As shown in Fig. 2 mean differences between eGFRs of protective (TT or TC) and risk (CC) haplotype kidneys for consecutive time points (3rd, 6th, 9th 12 ^th^ post-transplant months) were: 5.7, 6.05, 5.1 and 5.14 ml/min.

**Table 5:** Results of multi factor analyses evaluating for the independent variables^a^predicting

A. Estimated filtration (MDRD) of kidney allograft during first post-transplant year				
Variable		ΔeGFR±SD		p
rs3752462	CT + TT vs CC	+ 4.60 ± 2.33		**0.050**
time	3rd to 12th months	−3.07 ± 0.81		**< 0.001**
rs37524628^a^time	interaction	–		0.920
Donor sex	M vs F	+ 6.07 ± 2.33		**0.010**
DGF	Y vs N	−8.42 ± 2.45		**< 0.001**
B. Occurrence of proteinuria in kidney allograft during first post-transplant year				
Variable		OR	95% CI	p
rs136211	GG vs AG + AA	2.22	1.18–4.37	**0.017**
time	3rd to 12th months	0.99	0.65–1.49	0.313
rs136211^a^time	interaction	–	–	0.520
Donor sex	M vs F	0.45	0.22–0.91	**0.027**
Recipient sex	M vs F	1.76	0.92–3.39	0.087

^a^ data for variables with p ≤ 0.05 are shown in bold

**Fig. 3:**
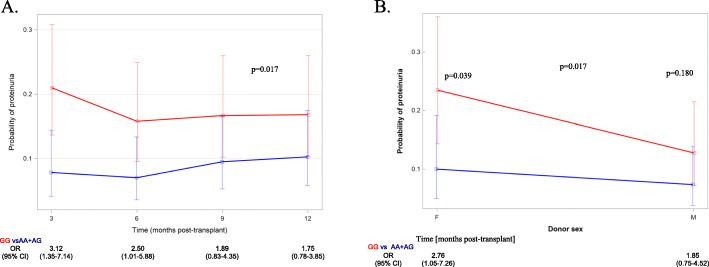
**a**. The risk of proteinuria within first post implantation year depending on rs136211 genotype (GG vs GA or AA, four time-points model) after adjustment for donor and recipient gender. **b.** The risk of proteinuria within first post-implantation year depending on donor gender after adjustment for donor rs136211 genotype (GG vs GA or AA) and recipient gender (F female, M male)

The multivariate prediction model of the risk of proteinuria included rs136211 GG genotype and donor and recipient sex (Table 5B), no time-dependent effect of GG genotype was found. As presented in Fig. 3a GG genotype at third and sixth post-transplant months significantly increased the risk of proteinuria, while at ninth and twelfth month this association turned statistically insignificant. Besides, we found that in female kidneys GG genotype resulted it 3-fold increased risk of proteinuria, while for male kidneys the difference in the risk of proteinuria was insignificant (Fig. 3b).

## Discussion

In previous studies, *MYH9* variants have been shown to be predictive for kidney function in the general adult population [10–12] as well as in progression of primary [20, 21] or secondary nephropathies [22–24]. Freedman et al. were the first to denote potential *MYH9* genotype impact on post-transplant FSGS relapse [25] while this study of ours investigates an association of *MYH9* variants with eGFR and proteinuria in a setting of kidney transplantation.

We found that donor *MYH9* rs3752462 C > T polymorphism predicted renal graft eGFR with-in 12 months follow up. Similarly, we found that donor *MYH9* rs136211A > G polymorphism predicted graft damage as expressed by increased risk of proteinuria.

These data seem to support a hypothesis that *MYH9* variation which is known to affect renal function in the general population also impacts engrafted kidneys.

The association of eGFR with rs3752462 T variant translated into an approximately 5 ml/min higher mean eGFRs within one-year of follow-up (Table 5A and Fig. 2). This is remarkably close to the effect sizes found for the well-established predictors of transplant survival with this estimate ranging from − 4.7 for additional 10 years of donor age above 25, − 3.3 ml/min for DGF, − 4.3 for recipient obesity, and + 3.3 for mycophenolic acid based immunosuppression, and it is half of the effect found for acute rejection episode − 9.2 ml/min lower graft filtration [18].

Alongside, it was well documented that eGFR below 50 ml/min in the first post-implantation year is a risk factor for further graft deterioration [26] and we have shown in our study group, that mean eGFRs achieved by rs3752462 risk (CC) genotype kidneys remained below this value, while those of protective genotypes (TT or TC) reached the mentioned threshold in the 6th post implantation month and steadily remained above 50 ml/min (Fig. 2b). Therefore, the clinical relevance of rs3752462 genotype for renal allograft filtration is potentially very high. It is further corroborated by the fact that rs3752462 contribution to graft function is independent of other final model predictors.

It includes delayed graft function as the independent variable potentially pointing at pathogenic pathway of rs3752462 in eliciting renal dysfunction. On the one side DGF is known to correlate with kidney ischemic injury [24], on the other ischemia severity (given as diminution of haemoglobin concentration) was shown to foster an association between the apolipoprotein 1 (*APOL1*) gene and *MYH9*-associated predispositions to sickle cell disease nephropathy [13, 14]. In a zebrafish model hypoxia evoked suppression of both the *MYH9* and *APOL1* genes and the similar nephrogenic effect was induced [13], while in sickle cell anemia mouse model increased kidney cortex *MYH9* expression was found [15]. These emphasize the prominent but functionally unclear role of ischemia on the *MYH-9* associated filtration variability.

Until now the T allele of rs3752462 was reported as a risk variant which in Caucasians correlated with lower glomerular filtrations [11, 23, 24, 27]. It is still unclear whether those findings reflected the mentioned SNP impact on non-muscle myosin heavy chain IIA expression, structure or function or, contrarily, whether they rather illustrate linkage with some other, functional but yet unknown, genetic variant. Our results show that in the post-transplant setting rs3752462 T allele revealed an opposite, protective effect on engrafted kidney filtration. This surprising result may indicate unique mechanisms (and distinct from those operating in native kidneys) mechanisms that underlie statistical correlation that we found. It is tempting to speculate whether those are linked to ischemic - reperfusion injury, exposition to immunosuppressives or arise due to allorecognition and rejection. While according to in vitro experiments non-muscle myosin heavy chain IIA regulates ischemia or oxidative stress-induced kardiomyocyte [28] and neuronal apoptosis [29], the issue of its involvement in ischemic kidney injury has not explored. On clinical grounds, our final model of GFR prediction showed that the effect of rs3752462 T allele was independent of DGF (which results from ischemia) and this implies independence of the SNPs impact from ischemia-reperfusion injury. Data regarding potential interplay between effects of immunosuppression and *MYH9* variants is very scarce. Corales and co [23] did not find an association between E1 haplotype (which includes rs3752462) and response to immunosuppressive treatment in lupus nephritis, whereas in nephrotic children one of *MYH9* polymorphisms correlated with tacrolimus concentrations although the authors did not elaborate on the treatment efficacy [30]. As all our patients received a combination immunosuppressive therapy including either tacrolimus or cyclosporine we could not evaluate whether this group of medications modifies rs3752462 impact on graft filtration.

Non-muscle myosin heavy chain IIA is known to be involved in immune system activation and its genetic variation might, potentially, modify autoimmune diseases course. Lin and co found an association of selected *MYH9* SNPs (although not rs3752462) with lupus nephritis occurrence [22], while in patients included in the already mentioned Corales study MYH8 SNPs did not correlate with lupus nephritis activity [23]. In potential analyses of *MYH9* variants relevance for engrafted kidney immune injury, recipient genotype should also be considered.

With regard to proteinuria, our findings indicate that the proteinuric effect of rs136211 may likely result from factors not related to recipient sex (one of predictors of final risk model of proteinuria, Table 5B). It is possible that this variant predisposes to or enhances proteinuria which may be triggered by hyperfiltration or pathologies occurring with-in the graft. According to our data (Fig. 3b) female kidney predisposes to *MYH9* associated proteinuria. As female gender is commonly associated with 12% lower number of nephrons [31], it may be inferred that resulting hyperfiltration may underlie proteinuric effect evoked by rs136211 risk genotype.

Moreover, this variant appears to act independently from other predictors of proteinuria [32–34]. This may also suggest that future advances in proteinuria management in recipients of kidney allografts may only be successful within the limit of genetic *MYH9*-associated predisposition.

Since proteinuria is a recognized predictor of graft and recipient survivals [33, 35] pretransplant donor genotyping may be potentially used to predict post-transplant course. One may speculate that older wait-listed patients could benefit from prompt transplantation with suboptimal organ (carrying nephrogenic risk variants) while younger ones could wait longer for kidneys that increase the chance of prolonged survival.

All evaluated SNP are located in intronic region of the *MYH9* gene and the mechanisms by which the risk variants potentially exert their effects on kidney function remains unknown. They may be of functional significance by influencing gene expression or pre-mRNA splicing or may tag functional mutations in other genes.

One hypothetical explanation of the role of *MYH9* variation in the progression of chronic nephropathies may be adopted from Keeling et al. [36]. According to this hypothesis keloids formation and glomerular sclerosis share similarities of pathogenesis and histology, with the presumed *MYH9* variation involvement. Since fibroblasts found in the growing part of keloids express excessive amounts of the NMMHC-IIA protein, it is possible that a similar process occurs in the kidneys [36]. In addition, NMMHC-IIA synthesis is stimulated by angiotensin II [37], known to have profibrotic properties and to be involved in the chronic injury of transplanted kidneys. Nonetheless, thus far, only few cases of coinciding keloids and CKD have been reported [38, 39] and there are also contradictory data concerning NMMHC-IIA expression in affected glomeruli, at least in certain proteinuria-associated nephropathies. According to morphological studies, NMMHC-IIA localizes in the podocyte cell body and primary processes; diminished expression of the latter was observed in primary focal segmental glomerulosclerosis, minimal change disease, and in a mouse puromycin aminonucleoside nephropathy model of acquired podocyte injury [40].

As recently stated by Zhao et al., some studies on variants of interest in individuals suffering from diabetes or kidney disease point at vascular abnormalities as a pathology linking nephrogenic alleles with CKD [41]. Rs3752462 was found to correlate with cerebral blood flows in individuals with Diabetic Kidney Disease [42], blood pressure control in patients with CKD [16] and in our previous study with transplanted kidney artery stenosis [43], all those potentially being markers of vessel wall genetic preponderance to injury or dysfunctional healing.

Our study should be viewed in the context of several limitations. First, due to the small sample size, there was a substantial baseline-characteristics diversity of donors and recipients. For this reason, we performed multivariate analyses to test for associations between genetic variants and graft dysfunction. Second, in order to eliminate acute events occurring early post-implantation that may interfere with the allograft function, assessment of filtration and proteinuria incidence started at third post-transplant month. Since we began patient observation after achievement of relatively stable graft filtration, we could not evaluate whether and how the genetic variants of interest may have affected graft function in the early post-transplant period (e.g., by predisposing to primary non-function). Third, while we identified associative relation between studied SNP and engrafted kidney function measures, the underlying phenomena, e.g. changed gene or protein expressions were not explored.

Finally, the relatively low incidence of certain genotypes found in our study (e.g., rs5756168-CC in 3 donors, 1.4%) may have likely limited the number of variants identified as significantly associated with filtration or proteinuria.

## Conclusion

In conclusion, we have demonstrated the relevance of *MYH9* variants for graft function within the first post-transplant year. Based on the presented results, a potentially high significance of *MYH9* variants for allografted kidney viability may be suggested. Overall, there are two distinct aspects of the results of our study: a theoretical one, related to the novelty of the genetic predictor of kidney allograft function; a practical aspect derives from the high clinical significance of *MYH9*-genotype and its strength being comparable to established clinical predictors of renal graft function. We believe that results of our study provide a rationale for prospective study evaluating donor *MYH9* genotype to predict graft function at 1 year after engraftment and graft survival.

## Supplementary information

**Additional file 1 : Supplementary Table 1.**

**Additional file 2 : Supplementary Table 2.**

**Additional file 3 : Supplementary Table 3.**

**Additional file 4 : Supplementary Table 4**. Chronic abnormalities of the transplanted kidney in high vs low risk variants of rs3752462 and rs136211.

**Additional file 5 : Supplementary Table 5.**

**Additional file 6: Supplementary figures.**

## Data Availability

The datasets used and analyzed during the current study are available from the corresponding author on reasonable request.

## Abbreviations

CKD: Chronic kidney disease
*MYH9*: Myosin-9 heavy chain gene
NMMHC-IIA: Nonmuscle myosin heavy chain II-A
SNP: Single nucleotide polymorphism
eGFR: Estimated glomerular filtration
HLA: Human leukocyte antigens
MDRD: Modification of diet in renal disease
PRA: Panel reactive antibodies
TIT: Total ischemia time
DGF: Delayed graft function
rATG: Rabbit antithymocyte globulin
IVIG: Intravenous immunoglobulins
ADPKD: Autosomal dominant polycystic kidney disease
GN: Glomerulonephritis
Csa: Cyclosporine A
Tacro: Tacrolimus
