# Supplementary material for: Effect of donor non-muscle myosin heavy chain (MYH9) gene polymorphisms on clinically relevant kidney allograft dysfunction
Source: BMC Nephrol. 2020 Sep 1;21:380. doi: 10.1186/s12882-020-02039-6 (PMC7465840; doi:10.1186/s12882-020-02039-6)
Supplement: Supplementary file 3 — Additional file 3 : Supplementary Table 3. [file 12882_2020_2039_MOESM3_ESM.docx]

|  | | **rs3752462** | | | | | **rs136211** | | | |
| --- | --- | --- | --- | --- | --- | --- | --- | --- | --- | --- |
| **clinical variable** | | **CT+TT** | **CC** | | **p** | | **AG+AA** | **GG** | **p** |  |
| Recipient sex F (%) | | 43.2 | 37.5 | | 0.477 | | 36.6 | 44.2 | 0.319 |  |
| **Recipient age (years)** | | **45.5 ±14.1** | **50.4±13.1** | | **0.007** | | 48.1±13.2 | 48.1±14.5 | 0.435 |  |
| Last PRA >20%, n (%) | | 18.3 | 11.6 | | 0.234 | | 6.4 | 10.5 | 0.318 |  |
| Retransplants, n (%) | | 24.2 | 16.1 | | 0.163 | | 17.9 | 22.1 | 0.487 |  |
| HLA mismatch, n (%)  0-3 | | 53.7 | 49.1 | | 0.577 | |  |  |  |  |
| DGF, (%) | | 13.5 | 20.6 | | 0.147 | | **14.1** | **20.0** | **0.024** |  |
| GN as primary kidney disease, (%) | | 41.0 | 39.3 | | 0.887 | | 42.9 | 36.8 | 0.397 |  |
| Preemptive Tx, n (%) | |  |  | |  | |  |  |  |  |
| Induction with ATG or aIL2, n (%) | | 29.8 | 33.0 | | 0.652 | | 30 | 33.3 | 0.651 |  |
| Cyclosporine A, n (%) | | 24.2 | 26.8 | | 0.750 | | 25.9 | 25.3 | 1.000 |  |
| Donor sex (woman), n (%) | 37.9 | | 42.3 | 0.570 | | 40.2 | | 40.4 | 1.000 |  |
| Donor age (years) | 43.4±13.1 | | 45.1±14.5 | 0.072 | | 44.2±13.0 | | 44.5±14.9 | 0.258 |  |
| Donor cause of death*, n (%) |  | |  |  | |  | |  |  |  |
| stroke | 60.0 | | 61.5 |  | | 65.0 | | 65.5 |  |  |
| trauma | 32.9 | | 36.4 |  | | 33.0 | | 37.0 |  |  |
| other | 7 | | 2 | 0.274 | | 2.0 | | 7.4 | 0.159 |  |
| pulsative perfusion, n(%) | 58.5 | | 63.4 | 0.477 | | 61.3 | | 61.0 | 1.000 |  |
| TIT** (hours) | **25.9** | | **27.6** | **0.04X** | | 26.4±8.4 | | 27.3±7.7 | 0.267 |  |
| Donor AKI | **47.4** | | **31.5** | **0.022** | | 39.3 | | 38.3 | 1.000 |  |
| AR 3 months | 16.8 | | 16.9 | 1.0 | | 12.5 | | 22.1 | 0.093 |  |
| AR 12 months | 26.3 | | 25.0 | 0.874 | | 22,3 | | 29.5 | 0.266 |  |
| GN post Tx | 3.2 | | 0 | 0.095 | | 0.9 | | 2.1 | 0.595 |  |

** missing data
