# Supplementary material for: Effect of donor non-muscle myosin heavy chain (MYH9) gene polymorphisms on clinically relevant kidney allograft dysfunction
Source: BMC Nephrol. 2020 Sep 1;21:380. doi: 10.1186/s12882-020-02039-6 (PMC7465840; doi:10.1186/s12882-020-02039-6)
Supplement: Supplementary file 4 — Additional file 4 : Supplementary Table 4. Chronic abnormalities of the transplanted kidney in high vs low risk variants of rs3752462 and rs136211. [file 12882_2020_2039_MOESM4_ESM.docx]

**Supplementary table 4.** Chronic abnormalities of the transplanted kidney in high vs low risk

variants of rs3752462 and rs136211

| histology variable  (% of specimens affected with pathology) | rs3752462 | | | rs136211 | | | |
| --- | --- | --- | --- | --- | --- | --- | --- |
|  | CT+TT | CC | p^#^ | AG+AA | GG | p^#^ |  |
| 0-30 days post Tx* | | | | | | |  |
| ah | 30.2 | 33.9 | 0.7006 | 34.3 | 29.1 | 0.5643 |  |
| ci | 3.2 | 3.3 | 1.0000 | 3.0 | 3.5 | 1.0000 |  |
| ct | 3.2 | 4.9 | 0.6772 | 3.0 | 5.3 | 0.6604 |  |
| cv | 36.5 | 47.5 | 0.2713 | 44.8 | 38.2 | 0.5803 |  |
| cg | 4.8 | 5.1 | 1.0000 | 4.5 | 5.4 | 1.0000 |  |
| 0-360 days post Tx** | | | | | | |  |
| ah | 36.2 | 42.4 | 0.4854 | 37.8 | 40.0 | 0.7266 |  |
| ci | 21.7 | 29.8 | 0.3289 | 24.3 | 27.4 | 0.6983 |  |
| ct | 21.7 | 34.3 | 0.1270 | 24.3 | 32.3 | 0.3409 |  |
| cv | 50.7 | 60.6 | 0.2994 | 55.4 | 55.7 | 1.0000 |  |
| cg | 7.2 | 4.5 | 0.7187 | 4.0 | 8.2 | 0.4672 |  |

* biopsy result available for 122 transplants

** biopsy result available for 136 transplants

^#^ Fisher's Exact Test
