# Supplementary material for: Effect of donor non-muscle myosin heavy chain (MYH9) gene polymorphisms on clinically relevant kidney allograft dysfunction
Source: BMC Nephrol. 2020 Sep 1;21:380. doi: 10.1186/s12882-020-02039-6 (PMC7465840; doi:10.1186/s12882-020-02039-6)
Supplement: Supplementary file 6 — Additional file 6: Supplementary figures. [file 12882_2020_2039_MOESM6_ESM.doc]

**Supplementary figures**

1. Mean eGFRs in the whole study group in the first post-transplant year (longitudinal observation)

Below we present the results based on raw data for eGFRs in the whole study group (the confidence limits are calculated assuming the normal distributions)

| **Analysis Variable : eGFR** | | | | |
| --- | --- | --- | --- | --- |
| **time** | **Mean** | **Std Error** | **Lower** | **Upper** |
| **3** | 46.91 | 1.14 | 44.68 | 49.15 |
| **6** | 49.75 | 1.22 | 47.36 | 52.15 |
| **9** | 50.25 | 1.19 | 47.92 | 52.57 |
| **12** | 50.58 | 1.24 | 48.15 | 53.00 |

Simple tests for differences between time points based on raw data would not take into account the auto-correlation effect and therefore should not be used here. That is why below we provide results based on calculations of relevant model and tests for differences in eGFRs in studied time-points in the table as well as figure.

Model without interaction effect

| **time Least Squares Means** | | | | |
| --- | --- | --- | --- | --- |
| **time** | **Estimate** | **Standard Error** | **Lower** | **Upper** |
| **3** | 45.68 | 1.32 | 43.09 | 48.26 |
| **6** | 48.10 | 1.31 | 45.52 | 50.69 |
| **9** | 48.44 | 1.32 | 45.85 | 51.03 |
| **12** | 48.76 | 1.31 | 46.18 | 51.34 |

| **Tests for changes in eGFR time between factor levels** | | | | | | |
| --- | --- | --- | --- | --- | --- | --- |
| **Effect** |  | **Estimate** | **Standard Error** | **DF** | **t Value** | **Pr > |t|** |
| **time** | **time={3,6}** | -2.43 | 0.80 | 499 | -3.02 | 0.0026 |
|  |  |  |  |  |  |  |
| **time** | **time={3,9}** | -2.76 | 0.81 | 499 | -3.42 | 0.0007 |
|  |  |  |  |  |  |  |
| **time** | **time={3,12}** | -3.08 | 0.80 | 499 | -3.84 | 0.0001 |

Supplementary Figure 1.


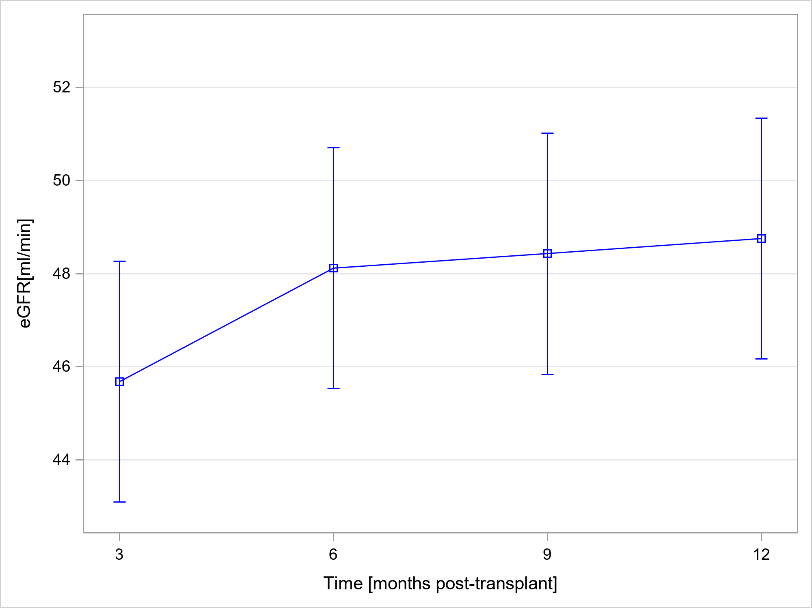


2. Proteinuria incidence in rs136211 GG *vs* AA+AG genotypes (univariant analysis)

| Fisher's Exact Tests for differences of Proteinuria fractions between 'AA+AG' and **'GG'** at individual time points | |
| --- | --- |
| **time** | **p-value** |
| 3 | 0.0161 |
| 6 | 0.0820 |
| 9 | 0.2200 |
| 12 | 0.3149 |

Supplementary Figure 2.

Simplified model assuming Poisson distribution for frequency:


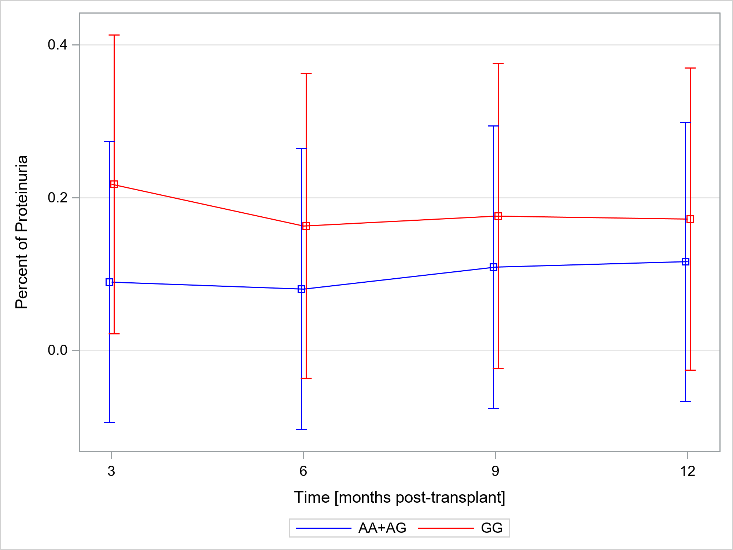


| **Percent based on raw data** | | | |
| --- | --- | --- | --- |
|  | | | **Assum. Poisson distribution for frequency** |
|
|
| **Group** | **time** | **Fraction** | **Std. error** |
| AA+AG | 3 | 0.0893 | 0.0938 |
|  | 6 | 0.0804 | 0.0939 |
|  | 9 | 0.1091 | 0.0943 |
|  | 12 | 0.1161 | 0.0933 |
| GG | 3 | 0.2174 | 0.0998 |
|  | 6 | 0.1630 | 0.1017 |
|  | 9 | 0.1758 | 0.1018 |
|  | 12 | 0.1720 | 0.1009 |
